# Metabolic and Cardiovascular Effects of Ghrelin

**DOI:** 10.1155/2010/864342

**Published:** 2010-03-16

**Authors:** Manfredi Tesauro, Francesca Schinzari, Miriam Caramanti, Renato Lauro, Carmine Cardillo

**Affiliations:** ^1^Department of Medicina Interna, Università di Tor Vergata, 00133 Rome, Italy; ^2^Istituto di Patologia Speciale Medica e Semeiotica Medica, Università Cattolica del Sacro Cuore, 00168 Roma, Italy

## Abstract

Ghrelin, an endogenous ligand for the growth hormone secretagogue receptor, is synthesized as a preprohormone and then proteolytically processed to yield a 28-amino acid peptide. This peptide was originally reported to induce growth hormone release; large evidence, however, has indicated many other physiological activities of ghrelin, including regulation of food intake and energy balance, as well as of lipid and glucose metabolism. Ghrelin receptors have been detected in the hypothalamus and the pituitary, but also in the cardiovascular system, where ghrelin exerts beneficial hemodynamic activities. Ghrelin administration acutely improves endothelial dysfunction by increasing nitric oxide bioavailability and normalizes the altered balance between endothelin-1 and nitric oxide within the vasculature of patients with metabolic syndrome. Other cardiovascular effects of ghrelin include improvement of left ventricular contractility and cardiac output, as well as reduction of arterial pressure and systemic vascular resistance. In addition, antinflammatory and antiapoptotic actions of ghrelin have been reported both in vivo and in vitro. This review summarizes the most recent findings on the metabolic and cardiovascular effects of ghrelin through GH-dependent and -independent mechanisms and the possible role of ghrelin as a therapeutic molecule for treating cardiovascular diseases.

## 1. Introduction

Ghrelin, a 28-amino-acid peptide hormone mainly secreted by the X/A-like cells in the oxyntic mucosa of the stomach, has been discovered as the endogenous ligand of the orphan receptor growth hormone secretagogues 1a (GHS-R1a) [1]. In addition to its marked growth hormone*** (***GH) releasing activity, ghrelin stimulates food intake and is involved in the regulation of energy homeostasis [2]. Furthermore, ghrelin has a variety of cardiovascular activities, including cardioprotective effects against ischemia, enhancement of vasodilation, cardiotropic effects, and regulation of blood pressure [3–5].

Human ghrelin gene is located on chromosome 3 and consists of 4 exons and 3 introns that encode a 117-amino-acid peptide, pre-proghrelin, that is cleaved into the mature 28-amino-acid form (3.3 kDa) secreted into the blood stream [6, 7]. Two major forms of ghrelin are found in tissues and plasma: *n*-octanoyl-modified ghrelin and des-acyl ghrelin [7]; ghrelin is the first known secreted bioactive peptide in mammals modified by an acyl acid on its third serine residues through the recently discovered enzyme ghrelin O-acyl transferase (GOAT) [8–10]. This postranslational modification is essential for binding to the GHS-R 1a and for several ghrelin biological activities, including the GH-releasing capacity and the actions on the endocrine axis, on energy balance and glucose homeostasis [3]. Nonacylated ghrelin, which represents the most abundant form of circulating ghrelin (80%–90%), has been found to be devoid of GH releasing capacity [1, 11, 12], but exerts a variety of physiological activity in the cardiovascular system as well as in lipid and glucose metabolism [13, 14]. 

Central and peripheral administration of des-acyl ghrelin significantly decreases food intake and decreases gastric emptying in food-deprived mice [15]. In another study, Chen et al. confirmed that des-acyl ghrelin decreases food intake in mice and showed that ghrelin disrupts the fasted motor activity of the antrum in freely moving conscious rats [16]. Moreover, centrally administrated des-acyl ghrelin increases feeding through activation of the orexin pathway and may act in hypothalamic feeding regulation [17].

Ghrelin plasma levels are mainly regulated by nutritional and metabolic factors; in fact they are increased by energy restriction (such as malnutrition, anorexia nervosa, and cachexia) and decreased by food intake and overfeeding [18]. These notions are in agreement with several studies showing reduced circulating ghrelin in patients with obesity and metabolic syndrome [19, 20]. Furthermore, ghrelin circulating levels increase in obese subjects when they lose weight [21, 22].

While all forms of human obesity have inappropriately low ghrelin levels, the only exception is the Prader-Willy syndrome, a complex genetic disorder characterized by mental retardation, hyperphagia, short height due to GH deficiency, and muscular hypotony [23]. In these patients, excessive appetite causes progressive obesity, which is surprisingly associated with high ghrelin levels in the same range of patients with anorexia nervosa.

In a recent study, Kirchner et al. suggested that the ghrelin/GOAT system acts as a lipid nutrient sensor that informs the central nervous system on the availability of substrates, rather than a meal initiation factor in reply to fasting [10]. It has also been reported that ghrelin is not inhibited by gastric distension due to water intake but is reduced by glucose administration [24]. Moreover, intravenous glucose loads inhibit ghrelin secretion, whereas protein intake seems to increase or to not modify ghrelin levels [25]. By contrast, constant infusion of lipids does not influence ghrelin levels, while oral administration of fats reduces them, even though not as much as glucose administration [26]. Moreover, it has been demonstrated that ghrelin induces an increase in glucose levels and this effect could be related to activation of glycogenolysis, increased liver gluconeogenesis, or stimulation of catecholamine release [27–30]. 

A negative association has been shown between ghrelin and insulin secretion [31], but the exact mechanisms by which insulin regulates ghrelin secretion need further studies. Hyperinsulinemia, however, has been suggested to act as a feedback mechanism to suppress ghrelin secretion, because several studies have reported reduced plasma ghrelin in association with different insulin resistance states, including hypertension, type 2 diabetes, or polycystic ovary syndrome [32].

A positive correlation between ghrelin and high-density lipoprotein (HDL) cholesterol concentration has also been observed [33, 34] and, in another study, Park et al. have shown that fasting plasma ghrelin levels are negatively correlated with triglycerides and positively correlated with HDL cholesterol in boys but not in girls; the mechanism underlying this sex difference, however, is not well established [35].

Ghrelin secretion seems to be under cholinergic control, since ghrelin levels are raised by cholinergic agonists and reduced by cholinergic antagonists [14]. In addition, Williams et al. showed that fast-induced rise of ghrelin levels is prevented by vagotomy and is reduced by atropine in rats [36]. Interestingly, ghrelin stimulates the differentiation of preadipocytes and antagonizes lipolysis in vitro [37] and promotes bone marrow adipogenesis in vivo [38]. Chronic ghrelin administration increases body weight, adiposity, and the expression of uncoupling protein (UCP) mRNA in brown and white adipose tissue in mice [39].

## 2. Tissue Distribution of Ghrelin and Ghrelin Receptor

Ghrelin is predominantly produced by the stomach, but it is also widely expressed in different tissues, such as the hypothalamus, pituitary gland, small and large intestine, placenta, pancreas, kidney, testes, ovary, and lymphocytes [40]. Ghrelin has also been found in several human neoplastic tissues and related cancer cells such as gastric and intestinal carcinoids, lymphomas and thyroid, breast, liver, lung, and prostate carcinomas [41, 42]. 

The ghrelin receptor (GHS-R) is a typical G-protein-coupled, seven transmembrane domain receptor. The gene of the human GHS-R 1a is located on chromosome 3q26.2 [43] and encodes for two different splice forms of the human GHS-R: GHS-R 1a, which binds ghrelin and leads to intracellular calcium mobilization, and GHS-R 1b, which is not able to bind ghrelin [44].

The GHS-R 1a is particularly concentrated in the hypothalamic-pituitary unit but is also present in other areas of the central nervous system and in several endocrine and nonendocrine tissues, including pancreas, lung, liver, kidney, small and large intestines, myocardium, spleen, ovary, testis, adrenal, adipose tissue, stomach, and the neuronal cells of the gut, both in animals and humans [45]. 

Also the GHS-R 1b has been found in many peripheral organs, including immune cells, skin, myocardium, pituitary, thyroid, pancreas, ileum, colon, liver, breast, spleen, duodenum, placenta, lung, adrenal, buccal mucosa, stomach, lymphonode, atrium, lymphocytes, and kidney [46]. Interestingly, both GHS-R subtypes have been found in tumoral tissues from organs that do not express these receptors in physiological conditions, for example, the breast [47].

In humans, the mRNA encoding GHS-R1a is detected in the cardiovascular system, but its expression in this system is much lower than in the pituitary [46]. Other authors have shown that human endothelial cells express ghrelin and the presence of ghrelin receptor has been reported in human endothelial cells, vascular smooth muscle cells, and the left ventricle [48]. Also GHS-R1b transcripts are highly expressed in human myocardium, but their physiological function is still unknown [46].

Analyzing ghrelin binding sites in the cardiovascular system, Katugampola et al. demonstrated a higher density of receptors in the myocardium of the right atrium than in the left ventricle, whereas aorta and pulmonary artery have more receptors than saphenous vein or coronary artery (Table 1). Moreover, the same investigators noticed a change in receptors density as a consequence of vascular diseases, with upregulation of ghrelin in vessels with advanced intimal thickening [49]. Binding studies have evidenced the existence of a subtype of ghrelin receptor distinct from GHS-R 1a, with the same binding affinity both for acylated ghrelin and nonacylated ghrelin, in H9C2 cardiomyocites and endothelial cells [50].

## 3. Cardiovascular Effects of Ghrelin

**Figure 1:**
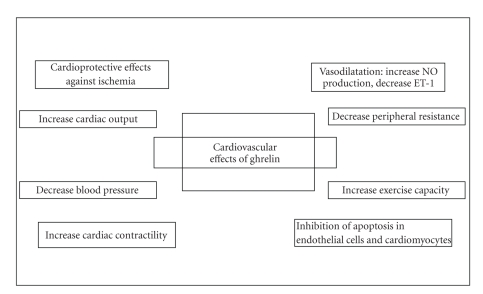
Cardiovascular effects of ghrelin.

Studies both in animal models and humans have shown beneficial effects of ghrelin in the cardiovascular system to support the wide expression of its receptors in cardiovascular tissues (Figure 1).

In animal models, ghrelin has been found to improve cardiac contractility in pathological conditions, to reduce the infarct size and to attenuate the reduction in left ventricular function induced by ischemia-reperfusion [51]. In particular, Frascarelli et al. have found that ghrelin administration significantly reduces the infarct size, as estimated by triphenyltetrazolium chloride staining, in a rat ischaemia-reperfusion model [52, 53]. Furthermore, in a group of rats following myocardial infarction, ghrelin administration has shown to increase body weight, cardiac output, and diastolic thickness of the noninfarcted posterior wall, as well as to inhibit left ventricular enlargement [53]. In animal models of heart failure, ghrelin administration improves cardiac contractility and attenuates the development of cardiac cachexia [52, 53]. 

In normal subjects, intravenous or subcutaneous ghrelin injection increases cardiac output, improves cardiac contractility, and causes a significant decrease in mean arterial pressure, without changing heart rate [54]. In addition to this, abundant evidence demonstrates a therapeutic effect of ghrelin in patients with heart failure. Thus, ghrelin has been reported to improve left-ventricular function and to attenuate left-ventricular remodelling in patients with chronic heart failure; in addition, acute ghrelin administration has shown to decrease systemic vascular resistances and increase cardiac output, cardiac index, and stroke volume index in patients with chronic heart failure [55]. The same authors have noticed that treatment with ghrelin for three weeks increases body weight, lean body mass, and muscle strength [55]. These results suggest that ghrelin could improve muscle wasting in patients with chronic heart failure and cardiac cachexia, a severe catabolic state characterized by weight loss and muscle wasting, resistant to long-term treatment with nutritional supplements.

In keeping with these findings, other studies have emphasized that ghrelin might have a role in patients with end-stage heart failure and cardiac cachexia, by improving cardiac function and increasing appetite [56, 57]. Ghrelin intravenous administration has therefore been proposed as adjuvant therapy in heart failure, due to its capacity to lead to a gain in left ventricular mass, to increase left ventricular ejection fraction, and to decrease left ventricular end-systolic volume [55].

A negative correlation has been noticed between ghrelin plasma levels and blood pressure, which might suggest that ghrelin is also involved in the sympathetic regulation. In fact, ghrelin seems to suppress sympathetic activity and to decrease blood pressure through mechanisms involving the central nervous system [58]. This hypothesis is supported by studies showing that ghrelin administration significantly decreases plasma norepinephrine levels and the ratio between low-to-high frequency spectra of heart rate variability in rats with myocardial infarction [59]. The reduction in peripheral resistance following ghrelin intravenous infusion results in a decrease in blood pressure levels in humans, even though supraphysiological hormone levels are required to produce this effect [54, 60]. Other mechanisms have been proposed to explain the reduction in blood pressure after ghrelin administration, including vasodilation via endothelium activation or a direct effect on vascular smooth muscle cells [27, 61].

In vitro studies suggested that, in human mammary artery, ghrelin causes vasorelaxation by antagonizing the endothelin-induced contraction [61]. We have demonstrated that ghrelin reverses endothelial dysfunction in patients with metabolic syndrome by increasing nitric oxide (NO) bioavailability [62]. We have also reported the molecular vascular actions of ghrelin, which stimulates the production of NO using a signaling pathway involving GHS-R 1a, PI 3 kinase, AKT, and eNOS [63]. More recently, we have extended our knowledge regarding the favorable endothelial actions of ghrelin by demonstrating that this peptide normalizes the altered NO/ET-1 balance within the vasculature of patients with metabolic syndrome, thus suggesting an important role of ghrelin in the regulation of vascular homeostasis [64].

## 4. Antiapoptotic Effects of Ghrelin in the Cardiovascular System

Ghrelin has also shown to act as an antiapoptotic peptide in the cardiovascular system. 

In vitro studies suggest that ghrelin stimulates H9c2 cardiomyocyte proliferation and reduces doxorubicin-induced apoptosis in cardiomyocytes and endothelial cells [50, 65]. Iglesias et al. found that ghrelin is synthesized and secreted by isolated murine and human cardiomyocytes and is able to prevent apoptosis induced by treatment with the apoptosis-inducer cytosine arabinoside (AraC) in mouse adult cardyomyocites cell line HL-1 [66]. Moreover, ghrelin treatment of primary cardiomyocytes prevents apoptosis stimulated by anti-FAS agonist antibodies. In addition, ghrelin stimulates tyrosine phosphorylation and activates ERK-1/2 and Akt in cardiomyocytes and endothelial cells; the activation of these two pathways is required for the antiapoptotic effect of ghrelin [50]. Furthermore, Isgaard et al. have found that ghrelin is also able to stimulate proliferation of H9c2 cardiomyocytes (a cardiac cell line that does not express the ghrelin receptor) in a dose-dependent and specific manner by increasing thymidine incorporation; however, they have reported the presence of alternative ghrelin binding sites on cardiomyocytes cell membranes in the absence of GHS-R 1a [67]. In another study, Kola et al. have proposed that ghrelin might modulate intracellular energy balance in a cell specific manner: ghrelin would be able to stimulate the 5′-AMP activated protein kinase (AMPK) that has a central role in regulating energy provisions in cells especially during anaerobic conditions [68]. Ghrelin effect on AMPK activity may be involved in the mechanism of cardioprotection from cellular injury and from ischemia-reperfusion damage [60].

## 5. Anti-inflammatory Effect of Ghrelin

Recent findings suggest that ghrelin has potent anti-inflammatory effects within the immune system and in human endothelial cells.

Dixit et al. found that ghrelin exerts specific and selective inhibitory effects on the expression of the inflammatory cytokines IL-1*β*, IL-6, and TNF-*α* [69]. Moreover, T lymphocytes express both ghrelin and the GHS-R and ghrelin secretion is increased when T lymphocytes are activated [69]. 

Administration of the GHS.1a agonist GHRP-2 results in decreased IL-6 levels and reduced signs of joint inflammation in arthritic rats [70]. Moreover, DeBoer et al. reported a significant decrease in circulating proinflammatory cytokines in ghrelin-treated rats with chronic kidney diseases (CKD); they also observed an increase in the circulating levels of the anti-inflammatory cytokines IL-10 in CKD rats treated with the ghrelin receptor agonist BIM-28125 [71]. In addition, ghrelin has potent anti-inflammatory effects in human endothelial cells, likely mediated by inhibition of NF-*κ*B, and also inhibits inflammatory cytokines produced by endothelial cells in response to LPS [72]. These anti-inflammatory effects of ghrelin suggest a possible modulatory role of the peptide as a novel strategy in several cardiometabolic disorders associated with chronic systemic inflammation.

## Figures and Tables

**Table 1:** GHS1 receptors in the human cardiovascular system.

Myocardium
Left ventricle
Aortic endothelium
Mammary artery
Coronary artery
Ventricular cardiomyocites
Vascular smooth muscle
Carotid
Saphenous vein
